# Diagnostic capability of CMR for the diagnosis of acute myocarditis in young patients is determined by the presence of elevated cardiac enzymes

**DOI:** 10.1186/1532-429X-14-S1-P183

**Published:** 2012-02-01

**Authors:** Tim G Schäufele, Sabine Rösch, Ina K Wenzelburger, Udo P Sechtem, Ali Yilmaz

**Affiliations:** 1 Cardiology, Robert-Bosch-Krankenhaus, Stuttgart, Germany

## Summary

Only in patients with elevated levels of cardiac troponin CMR can reliably corroborate the clinical diagnosis of acute myocarditis

## Background

Diagnosis of myocarditis remains a difficult clinical challenge, since the clinical spectrum of myocarditis is multifaceted. As clinical presentation and ECG-changes tend to be non-specific, cardiovascular magnetic resonance (CMR) has proven to be a valuable diagnostic tool regarding non-invasive diagnosis of myocarditis. However, CMR studies are time- and cost-intensive. Hence, further parameters are warranted to identify those individuals in whom a CMR study is likely to add crucial information regarding correct and timely diagnosis of acute myocarditis. The current study sought to elucidate the diagnostic yield of CMR in young patients aged ≤ 40 yrs with clinical diagnosis of myocarditis based on symptoms and ECG in relation to the presence or absence of elevated levels of cardiac troponin as an additional marker of acute myocardial injury.

## Methods

Between 2009 and 2011, young patients aged ≤ 40 yrs presenting with acute or subacute chest pain and significant new-onset ST-segment changes suggestive of myocarditis, prospectively underwent CMR studies after obstructive coronary artery disease (CAD) was ruled out by coronary angiography if risk factors were present. Black-blood T2-weighted turbo-spin-echo (TSE) sequences were obtained to detect myocardial edema. Late gadolinium enhancement (LGE) imaging was performed in order to detect focal areas of contrast enhancement. Studies were performed on a 1.5 Tesla MR-Scanner (Siemens Sonata).

## Results

During the recruitment period, 106 consecutive patients aged 17 to 40 yrs were included. Mean age was 29±7 yrs. In 19 patients additional cardiac catheterization was performed in order to definitely rule out obstructive CAD. LGE was successfully performed in all patients while in one patient T2-weighted edema images could not be obtained due to insufficient image quality. 13 (12%) patients showed significantly elevated levels of cardiac troponin. 15 (14%) patients showed focal late gadolinium enhancement (LGE) indicating myocarditis. All patients (100%) who presented with elevated levels of troponin showed presence of focal LGE. T2-weighted edema imaging showed correlating myocardial edema in 7 of these 13 individuals (54%). However, none of the 2 patients demonstrating LGE in the absence of elevated cardiac enzymes had signs of myocardial edema assessed by T2-weighted edema imaging but both turned out to have anamnestic evidence of subsided myocarditis in the past requiring exclusion of “acute” myocarditis. Moreover, the remaining 91 young patients with an unequivocal clinical diagnosis of myocarditis - but normal troponin levels - demonstrated normal findings by both T2-weighted edema and LGE imaging.

## Conclusions

In younger patients (≤ 40 yrs) presenting with symptoms and ECG changes suggestive of acute myocarditis, CMR based on T2-weighted edema and LGE imaging can reliably corroborate the clinical diagnosis of acute myocarditis - but only in the presence of elevated levels of cardiac enzymes.

## Funding

No specific funding.

